# Comparison of virulence-related determinants between the ST59-t437 and ST239-t030 genotypes of methicillin-resistant *Staphylococcus aureus*

**DOI:** 10.1186/s12866-021-02329-5

**Published:** 2021-10-02

**Authors:** Feng Liao, Wenpeng Gu, Xiaoqing Fu, Bin Yuan, Yunhui Zhang

**Affiliations:** 1 Faculty of Life Science and Technology, Kunming University of Science and Technology, Kunming, 650500 People’s Republic of China; 2 The Affiliated Hospital of Kunming University of Science and Technology, Kunming, 650500 People’s Republic of China; 3 Department of Respiratory Medicine, The First People’s Hospital of Yunnan Province, 650022 Kunming, People’s Republic of China; 4 Department of Acute Infectious Diseases Control and Prevention, Yunnan Provincial Centre for Disease Control and Prevention, 650022 Kunming, People’s Republic of China

**Keywords:** Methicillin-resistant *Staphylococcus aureus*, ST239-t030, ST59-t437, Phenotypic, Pathogenic ability

## Abstract

**Background:**

Methicillin-resistant *Staphylococcus aureus* (MRSA) is an important pathogen for human infection. Hospital-acquired (HA) and community-acquired (CA) MRSA infections are serious clinical problems worldwide. In this study, we selected typical HA-MRSA strain and CA-MRSA isolates from our previous research and compared their phenotypic and pathogenic abilities both in vitro and in vivo.

**Results:**

ST59-t437-SCC*mec*IVa (YNSA7) and ST59-t437-SCC*mec*Vb (YNSA53) belonged to two prevalent subclones of CA-MRSA, while ST239-t030-SCC*mec*III (YNSA163) was an HA-MRSA epidemic clone in Southwest China. ST59-t437 strains demonstrated faster growth ability, higher survival rate resistance to human blood, and more toxin secretion levels and cytotoxicity than ST239-t030. The virulence and regulatory genes of hld, psm-α, RNAIII, agrA, and crtN were highly expressed on CA-MRSA isolates, especially the ST59-t437-SCC*mec*IVa subclone. However, the ST239-t030 strain had the strongest adhesion and biofilm ability among these MRSA bacteria. Animal experiments revealed the most serious lethal effect on BALB/c mice caused by the YNSA7 strain infection. The survival rates of BALB/c mice infected with the three MRSA strains were 16.7, 50.0 and 100.0% for YNSA7, YNSA53 and YNSA163, respectively. Histopathological analyses of infected animals indicated that the lungs were the most seriously damaged organs, especially for ST59-t437 MRSA. Severe inflammatory reactions, tissue destruction, and massive exudation of inflammatory mediators and cells could be identified in ST59-t437 strain-infected animals.

**Conclusions:**

In general, ST59-t437 strains showed higher pathogenic ability than the ST239-t030 isolate, while ST239-t030 MRSA revealed the features prevalent in hospital settings, specifically for adhesion and biofilm ability.

## Background

Recently, the incidence of methicillin-resistant *Staphylococcus aureus* (MRSA) has increased globally [1]; MRSA has become the most important pathogen of hospital-acquired (HA) or community-acquired (CA) infections and is a serious clinical problem worldwide [2, 3]. This pathogen has caused huge public health burdens in Europe, America, Asia and China, especially for HA infections [4]. Because of the higher mortality rate due to systemic MRSA infections, characterizing isolates with regard to their genetic relatedness as well as their antimicrobial resistance profiles and major virulence factors to assess potential risks for patients has become particularly important.

The major molecular typing methods for *S. aureus* are pulsed-field gel electrophoresis (PFGE) [5], multilocus sequence typing (MLST) [6], *spa* typing and staphylococcal cassette chromosome *mec* (SCC*mec*) typing [7]. PFGE is considered a good method for outbreak investigation of *S. aureus*. MLST (ST type) is suitable for analysing the evolutionary relationship and clonal complexes of strains. Other methods also show high discriminatory abilities and usage. In addition, the complete genome sequencing method has become the gold standard for analysing the genomic diversity of bacteria [8]. At present, MRSA is a pandemic worldwide, showing different clones of strain dissemination, such as HA-MRSA from the 1960s and CA-MRSA clones from the middle 1990s. Since 1959, different MRSA clones have emerged in different regions, and the most frequently reported clones are clonal complexes (CCs) 5, CC8, CC22, CC30 and CC45 [9]. Among them, CC5 and CC8 are the primary clones, and ST239 and ST5 are both prevalent HA-MRSA clones. In addition, the most prevalent clone of CA-MRSA in Asia is ST59, although it is not the epidemic clone in other parts of the world [4].

Our previous study revealed that ST59-t437 and ST239-t030 were the major genotype profiles of MRSA for human infections in Southwest China [10]. The ST239-t030 strain belonged to the HA-MRSA clone, while ST59-t437 showed heterogeneity in provoking different clinical diseases in the community. The phylogenetic tree indicated that ST239-t030 isolates were more closely related to the T0131 strain from Tianjin, China, belonging to the ‘Turkish clade’ from Eastern Europe; two clusters of ST59-t437 clones of MRSA in Southwest China were generated, belonging to the ‘Asian-Pacific’ clone (AP) and ‘Taiwan’ clone (TW).

MRSA has many pathogenic mechanisms [11, 12]. However, different strains have different pathogenic abilities to produce toxins and resistance to host defence. Some virulence determinants are related to certain clonal types; therefore, it is important to analyse the phenotypic or pathogenic ability of specific clones of MRSA. In this study, we selected typical ST239-t030 (HA-MRSA) and ST59-t437 genotype isolates (CA-MRSA) from our previous research [10] and compared the phenotypic and pathogenic abilities among these strains both in vitro and in vivo.

## Results

### General information

The two CA-MRSA strains used in this study were isolated from young patients under 5 years old from the Department of Paediatrics, and both cases had pneumonia. The sample types of the isolated strains were nasopharyngeal aspirate and sputum. The YNSA7 isolate showed the ST59-t437-SCC*mec*IVa genotype, and YNSA53 was ST59-t437-SCC*mec*Vb, as shown in Table 1. The HA-MRSA strain was ST239-t030-SCC*mec*III type, isolated from an old hospitalized case from the Department of Respiratory Medicine, and the strain sample type was sputum. All these cases were cured and discharged after clinical treatments.

**Table 1:** General information about the MRSA strains used in this study

Lab number	Genotype	Age (years)	Gender	Clinical symptoms	Sample types	Department	Outcome	GenBank accession
YNSA7	ST59-t437-SCC*mec*IVa	2	Female	Pneumonia and septicemia	Nasopharyngeal aspirate	Paediatrics	Discharged	VCEM00000000
YNSA53	ST59-t437-SCC*mec*Vb	4	Male	Fever, cough, and pneumonia	Sputum	Paediatrics	Discharged	VCEZ00000000
YNSA163	ST239-t030-SCC*mec*III	68	Female	Fever and pneumonia	Sputum	Respiratory	Discharged	VCFW00000000

### Antibiotic resistance and virulence genes annotation

Both ST59-t437 strains in this study showed identical antibiotic resistance profiles, and all were sensitive to gentamicin (GEN), rifampicin (RIF) and vancomycin (VAN). However, the ST239-t030 isolate revealed different resistance results from ST59-t437 MRSA, as shown in Table 2. The possession of the virulence genes indicated that both ST59-t437 and ST239-t030 MRSA had many virulence genes encoding several toxins, such as nuclease, metalloprotease, serine protease, enterotoxins, staphylokinase and pore-forming toxin (Table 2).

**Table 2:** Antibiotic resistance and virulence gene annotations for the MRSA strains used in this study

Strains	Antibiotic resistance													Virulence genes
	LZD	CM	CIP	EM	GEN	LEV	OXA	PCN	RIF	SXT	TET	VAN	MXF	
YNSA7	R	R	I	R	S	R	R	R	S	R	R	S	R	*sak*, *scn*, *aur*, *hlgA*, *hlgB*, *hlgC*, *seb*, *sek*, *seq*, *lukF*, *lukS*
YNSA53	R	R	I	R	S	R	R	R	S	R	R	S	R	*scn*, *aur*, *hlgA*, *hlgB*, *hlgC*, *seb*, *sek*, *seq*, *lukF*, *lukS*
YNSA163	S	S	R	R	R	R	R	R	I	S	S	S	R	*aur*, *splA*, *splB*, *sak*, *scn*, *hlgA*, *hlgB*, *hlgC*, *lukD*, *lukE*, *sea*, *sek*, *seq*, *lukF*, *lukS*

Note: R, resistance; I, intermediate; S, sensitive

### Growth curve of the strains

**Fig. 1:**
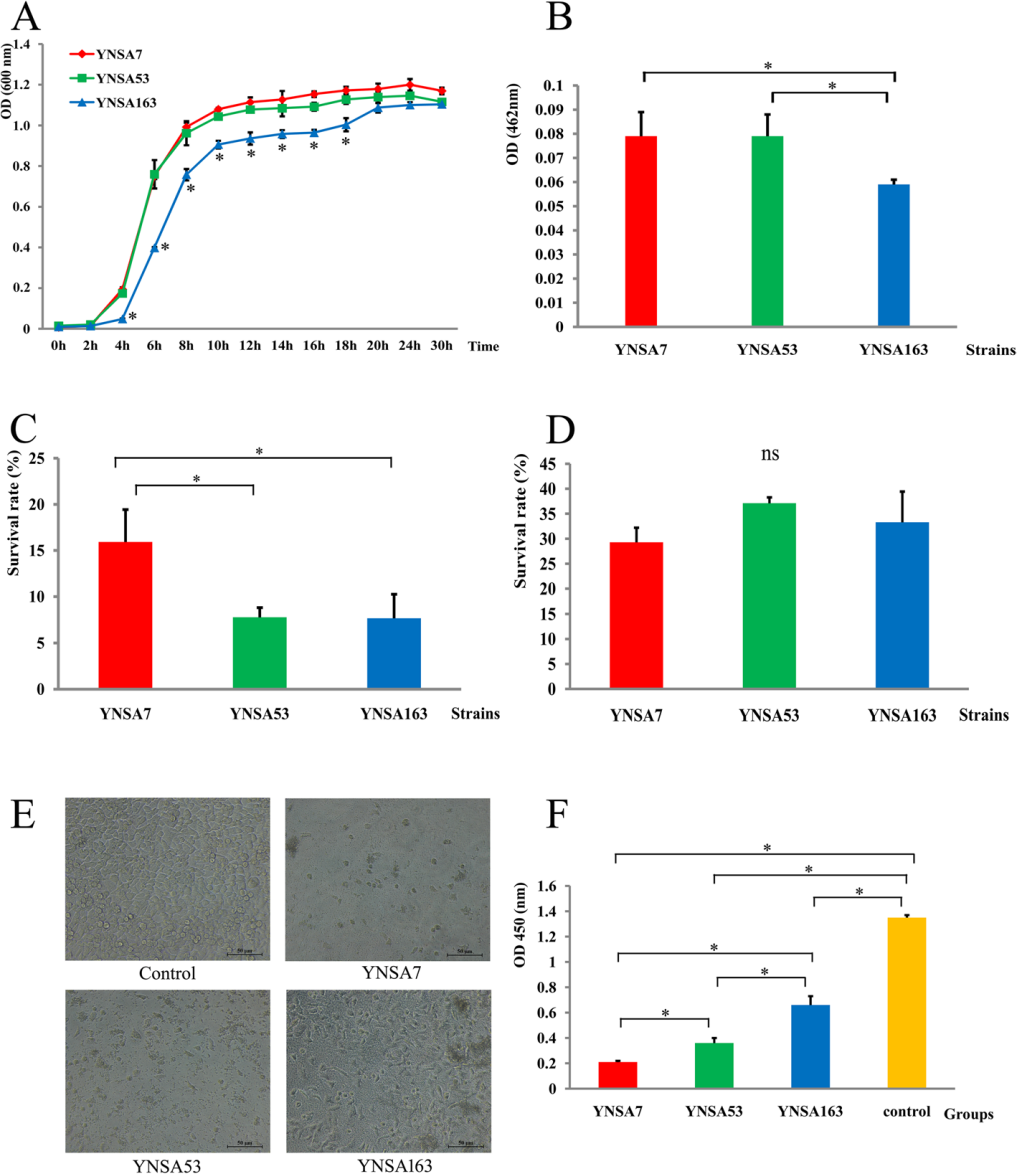
Phenotypic and cytotoxicity assays between the ST59-t437 and ST239-t030 strains in this study. *Statistically significant difference (*p* value < 0.05), and “ns” indicated no statistical significance. **A**. The growth curve of three MRSA strains; **B**. The staphyloxanthin results of three MRSA strains; **C**. Whole blood survival test of three MRSA strains; **D**. H_2_O_2_ killing examination of three MRSA strains; **E**. The light microscopy results of three MRSA-infected Hep2 cells (200×). The lab numbers in the figure indicated that the cells were infected with each strain; **F**. Cytotoxicity results of three MRSA strains infected with Hep2 cells

Comparison of the growth curves of the ST59-t437 and ST239-t030 strains indicated that the YNSA7 and YNSA53 isolates grew faster than the YNSA163 strain before 24 h in vitro, especially during the log phase. Statistical significance was found at 4, 6, 8, 10, 12, 14, 16 and 18 h between the two ST59-t437 strains and the ST239-t030 isolate, as shown in Fig. 1A.

### Staphyloxanthin assay

The staphyloxanthin assay revealed that the YNSA7 and YNSA53 strains produced more staphyloxanthin than YNSA163 (F = 15.279, *P* = 0.000), while no significant difference was found between the two ST59-t437 isolates by using the LSD method of the post hoc test in this study (Fig. 1B). Briefly, YNSA7 showed no statistical significance with YNSA53 (*P* = 0.907), while YNSA7 and YNSA53 were significantly different from YNSA163 (P = 0.000).

### Whole blood survival test

After bacteria were incubated with healthy whole blood from humans, 15.92 ± 3.51% of the YNSA7 strain survived after the experiment, and the survival rate for YNSA53 after incubation was 7.79 ± 1.05% and 7.69 ± 2.57% for the YNSA163 strain. A higher survival rate of YNSA7 was identified compared with YNSA53 and YNSA163 (F = 10.030, *P* = 0.012), as shown in Fig. 1C.

### H_2_O_2_ killing examination

H_2_O_2_ killing results showed no statistical significance among the three MRSA strains in this study (F = 2.876, *P* = 0.133). The survival rates were 29.29 ± 2.92%, 37.1 ± 1.19% and 33.29 ± 6.15% for YNSA7, YNSA53 and YNSA163 after H_2_O_2_ killing, respectively (Fig. 1D).

### Cytotoxicity assays

Cytotoxicity assays demonstrated that most Hep2 cells were deformed, necrotic and shedding after infection with the YNSA7 and YNSA53 isolates, especially YNSA7. Some of the Hep2 cells survived after YNSA163 strain infection, but an obvious cytopathic effect was identified (Fig. 1E). The cell viability test revealed that three MRSA-infected cells had statistical significance compared with the normal control (H = 21.60, *P* = 0.000). YNSA7 showed the worst cell viability among the three strains, followed by YNSA53 and YNA163 (Fig. 1F).

### qRT-PCR results of genes

**Fig. 2:**
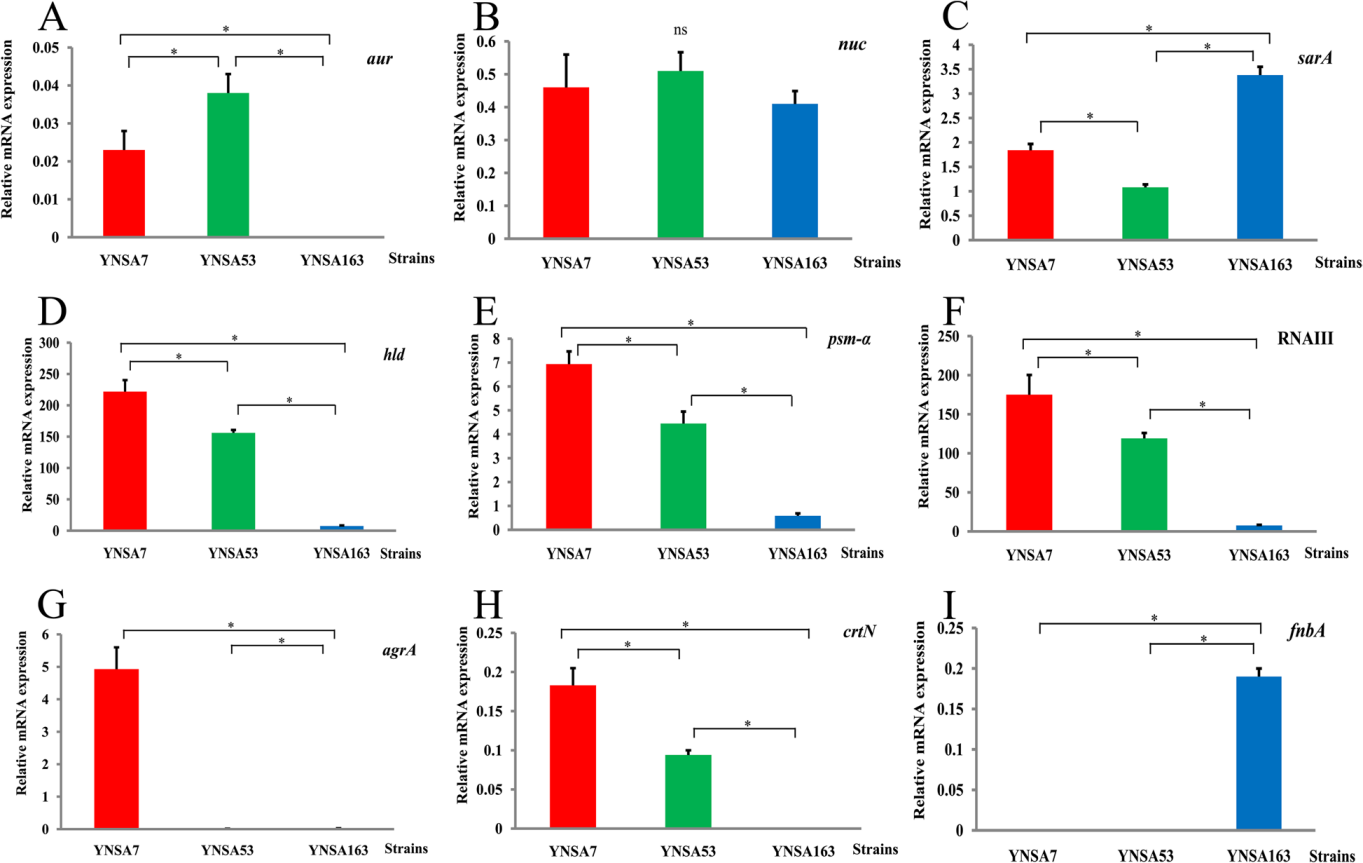
qRT-PCR results of virulence and regulatory genes of three MRSA isolates in this study. *Statistically significant difference (p value < 0.05), and “ns” indicated no statistical significance. **A**. aur gene; **B**. nuc gene; **C***. sarA* gene; **D**. hld gene; **E**. psm-α gene; **F**. RNAIII gene; **G**. agrA gene; **H**. crtN gene; **I**. fnbA gene

qRT-PCR results indicated that aur was highly expressed in the YNSA53 and YNSA7 strains compared with the YNSA163 isolate, as shown in Fig. 2A. In general, *aur* relative expression of YNSA53 showed a 38.0-fold change compared with YNSA163 and a 1.65-fold change compared with YNSA7; YNSA7 revealed a 23.0-fold change compared with the YNSA163 strain. There were no significant differences among the three MRSA strains for the nuc gene in this study (Fig. 2B). The relative expression of nuc showed 1.11- and 1.24-fold changes in the YNSA53 strain compared with the YNSA7 and YNSA163 strains, respectively; YNSA7 showed a 1.12-fold change compared with the YNSA163 strain. The sarA gene was highly expressed in the YNSA163 strain, followed by YNSA7 and YNSA53, as shown in Fig. 2C. The relative expression of sarA showed 1.84- and 3.13-fold changes in YNSA163 compared with the YNSA7 and YNSA53 strains, respectively; YNSA7 showed a 1.70-fold change compared with the YNSA53 strain. The YNSA7 isolate demonstrated high expression levels of the hld, psm-α, RNAIII, agrA, and crtN genes in vitro (Fig. 2D to H), while the YNSA163 strain exhibited the lowest expression levels of all these genes compared with the ST59-t437 strains in this study. Briefly, the relative expression of hld showed 29.60- and 20.81-fold changes in the YNSA7 strain compared with the YNSA53 and YNSA163 strains, respectively; YNSA53 showed a 1.42-fold change compared with the YNSA163 strain. The relative expression of psm-α showed 11.80- and 7.57-fold changes in the YNSA7 strain compared with the YNSA53 and YNSA163 strains, respectively; YNSA53 showed a 1.56-fold change compared with the YNSA163 strain. The relative expression of RNAIII showed 23.15- and 15.76-fold changes in the YNSA7 strain compared with the YNSA53 and YNSA163 strains, respectively; YNSA53 showed a 1.47-fold change compared with the YNSA163 strain. The relative expression of agrA showed 379.23- and 246.50-fold changes in the YNSA7 strain compared with the YNSA53 and YNSA163 strains, respectively; YNSA53 showed a 1.54-fold change compared with the YNSA163 strain. The relative expression of crtN showed 18.30- and 9.40-fold changes in the YNSA7 strain compared with the YNSA53 and YNSA163 strains, respectively; YNSA53 showed a 1.95-fold change compared with the YNSA163 strain. However, the fnbA gene was highly expressed in the YNSA163 isolate compared with the YNSA7 and YNSA53 strains (Fig. 2I). The relative expression of fnbA showed 19.30- and 19.80-fold changes in YNSA163 compared with the YNSA7 and YNSA53 strains, respectively; YNSA7 showed a 1.12-fold change compared with the YNSA53 strain.

### Biofilms assays

**Fig. 3:**
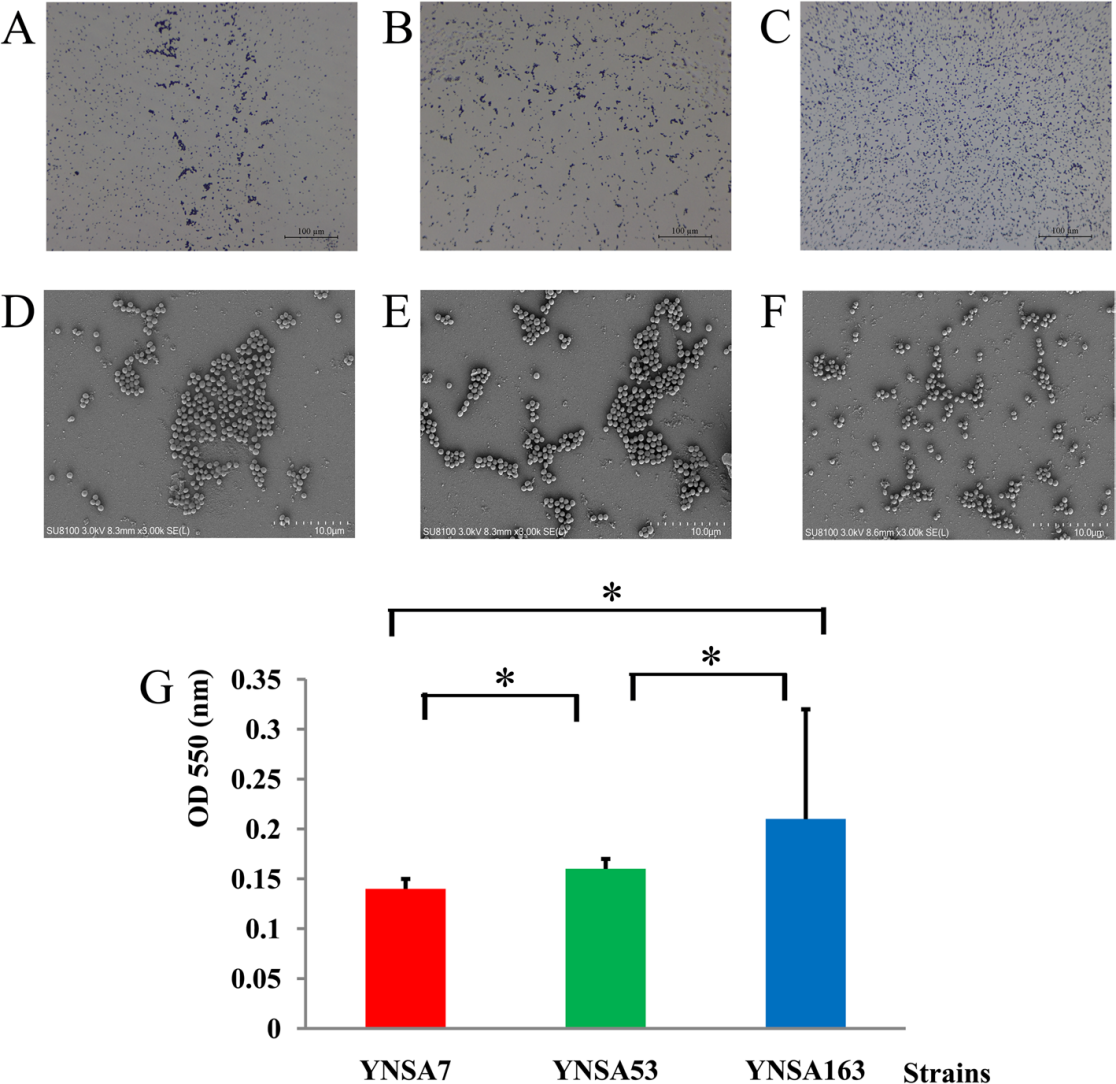
Biofilm assays of three MRSA strains in this study. **A**-**C** were light microscope examinations, and **D**-**E** were SEM examinations. **A**. Biofilm formed by YNSA7 (200×); **B**. Biofilm formed by YNSA53 (200×); **C**. Biofilm formed by YNSA163 (200×); **D**. Biofilm formed by YNSA7; **E**. Biofilm formed by YNSA53; **F**. Biofilm formed by YNSA163; **G**. Quantitative biofilm experimental results for the three MRSA strains. *Statistically significant difference (p value < 0.05)

The light microscopic examination results for the biofilms for three MRSA strains were shown in Fig. 3A to C. A large number of colonies of YNSA7 and YNSA53 adhered to the surface of plates (Fig. 3A and B), but the strongest adhesion ability of the strain was YNSA163 (Fig. 3C). The SEM results also revealed the biofilms of three MRSA isolates in this study, as shown in Fig. 3D to F. The masses formed by YNSA7 and YNSA53 were larger than those formed by YNSA163 (Fig. 3D and E), but the density of biofilms formed by YNSA163 was higher than those formed by YNSA7 and YNSA53 (Fig. 3F). The quantitative biofilm experimental results confirmed that the YNSA163 strain had the strongest adhesion ability among these MRSA isolates (F = 189.707, *P* = 0.000), followed by YNSA 53, and YNSA7 showed the lowest biofilm formation ability (Fig. 3G). The statistics were performed by using the LSD method for post hoc tests. In general, YNSA7 showed statistical significance with YNSA53 and YNSA163 (both *P* values were 0.000), and YNSA53 and YNSA163 also showed significant differences (P = 0.000).

### Animal experiments

**Fig. 4:**
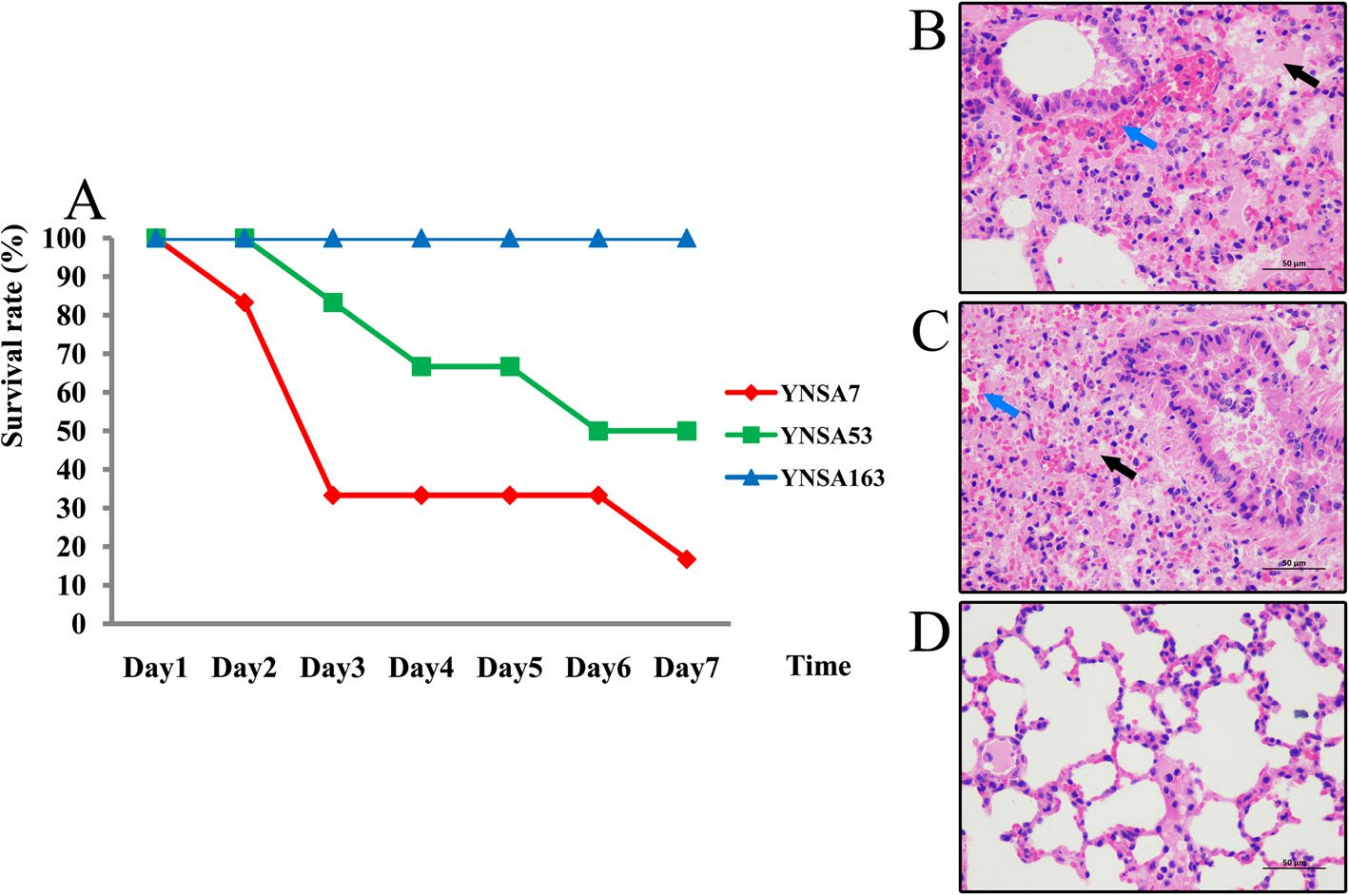
The animal experimental results for the three MRSA strain-infected BALB/c mice in this study. **A**. The survival rate of the three MRSA-infected BALB/c mice; **B**. Histopathological analysis of the lung for YNSA7 infection. The black arrow indicated widespread eosinophilic homogenate, and the blue arrow indicated a large amount of congestion; **C**. Histopathological analysis of the lung for YNSA53 infection. The black arrow indicated widespread eosinophilic homogenate, and the blue arrow indicated a large amount of congestion; **D**. Histopathological analysis of the lung for YNSA163 infection

The animal experiments revealed the most serious lethal effect on BALB/c mice caused by YNSA7 strain infection. At day 2 after bacterial injection, just one animal died; however, at day 3 of infection, three mice died. Finally, the survival rate of BALB/c mice infected with the YNSA7 isolate was 16.7% (Fig. 4A). Only one animal died on days 3, 4 and 6 after YNSA53 strain infection; thus, the survival rate of BALB/c mice was 50% by YNSA53 infection at the end of the experiment. No BALB/c mice died from YNSA163 isolate infection during the experimental process (Fig. 4A).

Histopathological analyses of the tissues and organs of infected BALB/c mice indicated that the lungs were the most seriously damaged organs. Extensive tissue necrosis, pyknosis or fragmentation of nuclei, and widespread eosinophilic homogenate (black arrow) could be found by infection with the YNSA7 strain (Fig. 4B). The structure of the alveoli was unclear, and a large amount of congestion was identified in blood vessels (blue arrow in Fig. 4B). For YNSA53-infected mice, similar pathological changes could be found in the YNSA7 group. HE examination revealed large areas of tissue necrosis, pyknosis or fragmentation of nuclei, and eosinophilic homogenate (black arrows) in YNSA53-infected lung tissues (Fig. 4C); the structure of the alveoli was also unclear, and large amounts of bleeding or congestion were seen in blood vessels (blue arrow). Compared with the ST59-t437-infected groups, there was no obvious abnormality in the structure of the bronchus at any level in the YNSA163 infection group. The alveolar wall was composed of a single layer of epithelium with a clear structure (Fig. 4D). The connective tissue and blood vessels in the lung had no abnormalities. In general, no obvious inflammatory changes were identified by ST239-t030 MRSA infection.

## Discussion

Several factors contribute to the success of MRSA as a pathogen and provoke the host’s innate and adaptive immune responses during infection [13]. Some secreted toxins, such as pore-forming toxins and superantigens, cause cytolysis and inflammation, coagulases and staphylokinases attack the host coagulation system, and nucleases and proteases inactivate the immune defence of the host [14]. MRSA strains could form biofilms on the host surface, evading host defences and antimicrobials. The biofilms were difficult to eradicate without removal of the intravenous catheter or medical device [11]. The phenotypic and functional results of the ST239-t030 isolate in this study showed lower pathogenic ability than the ST59-t437 strains, both in vitro and in vivo. However, higher adhesion and biofilm formation abilities were found for the ST239-t030 isolate, which could be partly explained by the prevalence in hospital environments. The strain could persist in the hospital setting and was difficult to remove due to its strong adhesion to the surface of the objects and medical equipment. In addition, the low pathogenicity of the strain allowed it to coexist with the host for a long time, causing the epidemic and dissemination of the clonal population [15]. Moreover, the antibiotic resistance profiles of ST239-t030 were different from those of the ST59-t437 strains, which revealed resistance to quinolone and aminoglycoside antibiotics. These drugs were commonly used in clinical treatment in hospital settings, which possibly reflected the establishment and spread of clones for HA-MRSA infection.

CA-MRSA usually causes soft tissue and skin infections, abscesses and folliculitis, especially in young patients [13]. For some CA-MRSA cases, fatal necrotizing pneumonia and necrotizing fasciitis could be identified [16, 17]. In this study, both ST59-t437 strains were recognized as CA-MRSA and isolated from children under 5 years old. The clinical manifestations of these cases were fever and pneumonia. All these features were similar to the characteristics of CA-MRSA infection. A study on CA-MRSA strains harbouring SCCmec types IV and V revealed that they replicated more rapidly than HA-MRSA isolates with other types of SCCmec types [18, 19]. The decreased transmission of larger structural elements of SCCmec types of strains might account for the lower number of HA-MRSA clones around the world [4]. In this study, we also revealed that ST59-t437 strains (CA-MRSA) grew faster than the ST239-t030 isolate (HA-MRSA) in vitro. Furthermore, ST59-t437 MRSA isolates showed more staphyloxanthin and cytotoxicity than the ST239-t030 strain in this study; a stronger resistance ability to human blood was also found in ST59-t437 compared with ST239-t030. All these pieces of evidence indicated that ST59-t437 (CA-MRSA) strains had a higher pathogenicity in vitro than the ST239-t030 isolate (HA-MRSA).

CA-MRSA clones have evolved in different geographic regions. ST8 is currently the most prevalent one in the United States [20, 21]. However, the most predominant clone of CA-MRSA in Europe is ST80, followed by ST5, ST8, ST30, and ST59 [22]. The most prevalent clone of CA-MRSA in Asia is ST59, although it is not the epidemic clone in other parts of the world [23, 24]. Chen et al. previously compared the features and pathogenic abilities of two epidemic CA-MRSA clones of the ST59 lineage in Taiwan, namely, the TW clone and AP clone [25]. The TW clone carried the PVL gene and SCCmec Vt type, while the AP clone was PVL negative and showed the SCCmec IV type. In their study, they concluded that the TW clone showed higher virulence in both humans and animal infection models, and they considered the acquisition of PVL, including other virulence factors that contributed to its enhanced pathogenic ability compared with the AP clone. Our previous study [10] verified that two subclones of the ST59 genotype of MRSA were prevalent in Southwest China, similar to Taiwan. In this study, both subclones of ST59-t437 isolates were used to compare their pathogenic abilities. However, the ST59-t437-SCC*mec*IVa clone showed higher pathogenicity than the ST59-t437-SCC*mec*Vb clone, and more toxin levels and serious lethality of animals could be found for the ST59-t437-SCC*mec*IVa clone. The gene annotations of previous research showed that both ST59-t437 isolates in this study had *PVL* genes. However, the transcriptome sequencing of three strains for differential gene expression (our unpublished data) revealed no significant difference in *PVL* gene expression between the two ST59-t437 MRSA strains in this study. Therefore, we considered that other factors might contribute to the enhanced pathogenic ability of the ST59-t437-SCC*mec*IVa strain.

The upregulated expression of hld, psm-α, RNAIII, and agrA genes on ST59-t437-SCC*mec*IVa might illustrate the higher pathogenicity of the strain. Regulations of gene expression play an important role in the pathogenesis of MRSA strains. The accessory gene regulator (agr) is a quorum sensing system that plays a critical role in the regulation of MRSA virulence. A previous study showed the decreased virulence found in agr mutant strains and certain types of agr led to specific clinical syndromes [26]. Phenol soluble modulins (PSMs) belong to an amphipathic peptide family in staphylococci, which have several roles in MRSA pathogenesis, including biofilm formation, cell lysis, and immune modulation [27]. For example, psm-α encodes peptides in lysing eukaryotic cells through cell membranes [28]. PSMs are encoded in three loci in the core genome of *S. aureus*: the *psm-α* operon, encoding PSMα1–4; the *psm-β* operon, encoding PSMβ1–2; and *hld*, encoding δ-toxin. *Hld* is also part of the coding sequence of RNA III, the master regulatory RNA in staphylococci [29]. All these genes were important to MRSA virulence, and they were all upregulated in the ST59-t437-SCC*mec*IVa clone compared with the ST59-t437-SCC*mec*Vb clone and ST239-t030-SCC*mec*III clone in this study.

In addition, pathological examination results showed that the strains mainly attacked the lungs of the host, especially the ST59-t437 strains, which caused severe inflammatory reactions, tissue destruction, and massive exudation of inflammatory mediators and cells. All these changes resulted in ventilatory and ventilation dysfunction of the host, leading to the death of the animal.

## Conclusions

In this study, we selected and compared the phenotypic and pathogenic abilities among prevalent clones of MRSA in Southwest China, including ST59-t437 and ST239-t030. ST59-t437 strains showed higher pathogenic ability than the ST239-t030 isolate both in vitro and in vivo, while ST239-t030 MRSA revealed the features prevalent in hospital settings, specifically for adhesion and biofilm ability. These results might partially indicate the reasons for the prevalence of different clones in environments and provoke different clinical infections.

## Methods

### Bacterial source, culture conditions and genomic sequencing

The MRSA strains used in this study were shown in Table 1. The bacteria were cultured at 37 °C on tryptone soya agar (TSA) (Thermo Scientific). Tryptone soya broth (TSB) (Thermo Scientific) was used for biofilm and virulence experiments. For the genomic sequencing of the isolates in this study, all MRSA isolates were recovered on TSA at 37 °C for 24 h. Total genomic DNA of the bacteria was extracted using a bacterial total genomic DNA extraction kit (Tiangen, Beijing) following the manufacturer’s instructions. Genomic sequencing was performed on the Illumina HiSeq platform by using 2 × 150 bp paired-end reads. The libraries were built using the Nextera XT DNA Library Prep Kit following the manufacturer’s reference guidelines (Illumina, USA) [10, 30]. The raw sequencing data were trimmed for quality control, and draft genomes were assembled using SOAPdenovo (version 2.04) with k-mer values optimized to the best assembly results [31, 32]. GenemarkS software (version 4.28) with default parameters was used to predict the open reading frame (ORF) of each genome, and the predicted amino acid sequences were aligned and annotated by DIAMOND (E-value: 1e-5, top 5) to the NCBI nonredundant nucleotide database (NR) [33]. The SCCmec types of isolates and virulence genes were detected by submitting the assembly genomes of strains to web-based SCCmecFinder and VirulenceFinder (http://www.genomicepidemiology.org).

### Antibiotic resistance detection

Thirteen antibiotics were determined through the broth microdilution method using customized microtiter plates (Sensititre, UK) according to the manufacturers’ instructions. The antibiotics tested were penicillin (PCN), oxacillin (OXA), gentamicin (GEN), ciprofloxacin (CIP), levofloxacin (LEV), moxifloxacin (MXF), erythromycin (EM), clindamycin (CM), linezolid (LZD), vancomycin (VAN), tetracycline (TET), rifampicin (RIF) and trimethoprim/sulfamethoxazole (SXT). The tests were performed and interpreted in accordance with the Clinical and Laboratory Standards Institute (CLSI) guidelines (M100, 2018), and *S. aureus* ATCC 29213 was used as a quality control [10].

### Growth curve

To compare the growth curve between ST239-t030 and ST59-t437 in this study, 20 mL of TSB was inoculated with 100 μL of MRSA strains cultured for 16 h (10^5^ CFU/mL bacterial suspension). The isolates were kept at 37 °C with shaking, and the OD values were measured at 600 nm every 2 h [34].

### Staphyloxanthin assay

Twenty mL of TSB was inoculated with 100 μL of a 16-h culture of MRSA strains (10^5^ CFU/mL bacterial suspension) and kept at 37 °C with shaking for 24 h. The bacteria were centrifuged at 12,000×g for 5 min, and the supernatants were removed. The pellets were resuspended by methanol extraction under shaken conditions at 37 °C overnight. The extracted staphyloxanthin was detected at OD 462 nm [35].

### Whole blood survival test

MRSA strains were grown on TSA medium, and 10^5^ CFU of isolates were adjusted by using saline. Bacteria were added to healthy human blood (heparin sodium was used as an anticoagulant) at a ratio of 1:4 and incubated at 37 °C for 3 h with shaking. The surviving isolates were calculated by the plate counting method as described previously [34, 36].

### H_2_O_2_ killing experiment

MRSA strains were grown on TSA medium, and 10^8^ CFU of isolates were adjusted by using saline. The bacterial suspensions were treated with 0.2% H_2_O_2_ at 37 °C for 3 h. Then, the plate counting method was used to analyse the viable surviving bacterial cells [37].

### Cytotoxicity assays

Monolayer Hep2 cells were prepared by seeding 96-well plates with 100 μL of cell suspension (10^5^ cells/mL) cultured with DMEM (Thermo Scientific) with glutamine supplemented with 10% foetal bovine serum (Thermo Scientific) at 37 °C containing 5% CO_2_ for 24 h. A concentration of 10^5^ CFU/mL strains was used, and 100-μL bacterial suspensions were added to Hep2 cells and then cocultured for 48 h at 37 °C. After incubation, the plates were washed with PBS buffer (pH 7.4) three times [38]. Cytotoxicity was determined by a CCK-8 kit according to the manufacturer’s instructions (Dojindo, Japan). Finally, the cells were examined by light microscopy (Nikon, Japan).

### Quantitative RT-PCR for virulence-related genes

MRSA strains were cultured for 16 h and diluted with TSB 100 times as described before. The cultures were incubated in a 37 °C shaker to logarithmic phase, and then bacteria were collected by centrifugation at 12,000×g for 5 min. Total RNA was extracted by using the TRIzol reagent (Ambion, Thermo Fisher). Expression analysis was performed using a CFX-96 real-time PCR instrument (Bio-Rad, USA) for sarA, aur, nuc, agrA, fnbA, RNAIII, hld, psm-α and crtN, referring to virulence and biofilm formation [34]. Real-time quantitative PCR, according to the instructions of the One Step TB Green PrimeScript PLUS RT-PCR Kit (TaKaRa, Japan), was used to analyse the relative mRNA expression of the genes. The 2-ΔCt method was used to calculate the relative mRNA expression results; gyrB was used as an internal control. The primers used for the gene expression analysis were based on a previous report [34].

### Biofilm assay

The biofilm assay was performed according to a previous study [39]. Briefly, bacteria were cultured for 16 h, diluted with TSB 100 times, and then seeded into 96-well plates (100 μL/well). The plates were incubated for 24 h at 37 °C, and then, culture supernatants were removed, followed by washing three times with PBS buffer (pH 7.4). Each well was fixed with 70% methanol for 30 min and dried. The biofilms were stained with 0.1% crystal violet (100 μL/well) for 30 min, washed with flowing water, and subsequently dried at 37 °C for 3 h. Finally, 100 μL of 95% ethanol was added to each well and vortexed. A wavelength of 550 nm was used to measure the absorbance.

For light microscopic examination, the washed plates were stained with 0.4% crystal violet, washed with PBS buffer to remove the excess stain, and then air-dried. Finally, the plates were examined under a light microscope at a magnification of 200× (Nikon, Japan).

### Scanning electron microscopy (SEM) of biofilms

MRSA strains were cultured for 16 h, diluted with TSB 100 times, and then seeded into 12-well plates containing sterile coverslips. The strains were cultured for 24 h at 37 °C, and then, the plates were washed three times with sterile PBS buffer (pH 7.4). The coverslips were fixed with 2.5% glutaraldehyde solution for 30 min at room temperature and washed with PBS buffer three times. Finally, the coverslips were dehydrated by an ethanol gradient (30, 50, 70, 80, 90, 95 and 100%), dried and ion plated, and then observed by SEM (SU8100, HITACHI) [34].

### Animal infection model

BALB/c mice were used for in vivo experiments and obtained commercially from the Institute of Medical Biology, Chinese Academy of Medical Sciences and Peking Union Medical College. The animals were divided into four groups, containing three MRSA isolate-infected groups and one control group. Each group had six animals. Mice were infected via intravenous injection with 2 × 10^8^ CFU of three MRSA strains. The control group of mice received PBS with the same volume of infected groups. The animals were observed daily for 1 week, and at 7 days after infection, the BALB/c mice were euthanized by cervical dislocation. Histopathological analyses were performed on the hearts, livers, spleens, lungs and kidneys of animals, and the tissues were fixed with 4% paraformaldehyde, stained and examined by using the HE method.

### Statistical analysis

Statistical analyses were performed using SPSS (version 16.0, IBM, USA). The Kolmogorov-Smirnov test, t-test, ANOVA, and Kruskal-Wallis H test was used as appropriate. A *P* value < 0.05 was considered to indicate statistical significance.

## Data Availability

The datasets generated and/or analysed during the current study are available in the NCBI database repository under BioProject accession number PRJNA543691 [https://www.ncbi.nlm.nih.gov/bioproject/PRJNA543691].

## Abbreviations

MRSA: methicillin-resistant *Staphylococcus aureus*
CA: community-acquired
HA: hospital-acquired
MLST: multilocus sequence typing
